# The Role of Modern Radiological Procedures in Diagnosing Blunt Liver Injuries Manifested by Upper Gastrointestinal Bleeding

**DOI:** 10.3390/jcm14010175

**Published:** 2024-12-31

**Authors:** Piotr Tomasz Arkuszewski, Maciej Adam Rybicki, Bartłomiej Białas, Konrad Szymczyk

**Affiliations:** 1 Department of Biomedicine and Experimental Surgery, Faculty of Medicine, Medical University of Lodz, Narutowicza 60, 90-136 Lodz, Poland; 2 Student’s Research Group, Department of Biomedicine and Experimental Surgery, Faculty of Medicine, Medical University of Lodz, Narutowicza 60, 90-136 Lodz, Poland; bartlomiej.bialas@stud.umed.lodz.pl; 3 Ist Department of Radiology and Diagnostics Imaging, Faculty of Medicine, Medical University of Lodz, Narutowicza 60, 90-136 Lodz, Poland; konrad.szymczyk@umed.lodz.pl

**Keywords:** blunt liver trauma, upper gastrointestinal bleeding, haemobilia, USG, CT, scintigraphy

## Abstract

**Objectives:** Posttraumatic upper gastrointestinal bleeding (UGIB) is a very rare consequence of blunt liver trauma. It can be quite a diagnostic challenge for clinicians, as it can clinically manifest many weeks after the trauma or be scantily symptomatic. **Methods:** The following article would like to provide an analysis of clinical cases of 13 patients following blunt liver injuries, the main symptoms of which was bleeding into the gastrointestinal tract through the biliary tree. The article is research of the published literature concentrating on the influence of modern diagnostic methods (scintigraphy, USG and CT) on the diagnosis and long-term survival of patients with haemobilia caused by blunt liver trauma. In each patient, the condition was presented with UGIB symptoms following blunt trauma, before initiation of operative treatment or before death. The cases were divided into 2 groups: prior to and after introduction of modern diagnostic procedures, and then compared together. **Results:** The study indicates that liver damage can cause symptoms of UGIB, even after minor abdominal trauma and with delayed and uncharacteristic symptoms. **Conclusions:** Modern diagnostic methods, such as ultrasound, scintigraphy and CT, make it easier to identify these injuries and choose appropriate treatment, reducing the risk of death.

## 1. Introduction

Liver injury following blunt abdominal trauma is observed in medical practice widespread. It typically manifests as hemoperitoneum and subcapsular or intraparenchymal hematoma [1,2,3,4]. However, post-traumatic liver injury presents in a specific and small number of cases with the symptom of upper gastrointestinal bleeding (UGIB) [5,6,7,8,9,10,11,12,13,14,15,16]. At least some of these medical conditions may be considered post-traumatic hemobilia. The difficulty in assigning individual cases is due to the ambiguous criteria for classifying hemobilia. In the most literal sense, hemobilia means the presence of blood in the bile ducts or bleeding into the billary tree. However, this does not determine that the source of the bleeding is the liver.

The presence of three different criteria for hemobilia are considered relevant in the professional literature:

- Quincke’s triad [17], i.e., pain in the right upper quadrant, upper gastrointestinal bleeding, and jaundice [18];
- Bleeding of arterial etiology [19,20];
- Bleeding of arterial or venous etiology from the liver through the biliary tract [20,21,22].

The last criterion was applied, in which the entire Quincke’s triad is not required. This is because the full triad occurs in only 22–35% of patients with hemobilia [17,21,23]. Also, in some cases analyzed by the authors, a pseudoaneurysm developed, which suggests an arterial etiology [12,14,15]. Therefore, some cases meet two or even all definitions of hemobilia. The classification criteria adopted allow for a broader consideration of cases and may better reflect the clinical diversity observed in practice. In fact, liver injury proceeding with bleeding into the bile ducts and further into the gastrointestinal tract represents post-traumatic hemobilia. In these situations, blood enters the duodenum from the traumatically ruptured liver, through the hepatobiliary tract. This explains the presence of typical symptoms of upper instead of lower gastrointestinal bleeding. The most characteristic symptoms of UGIB are hematemesis, melena, coffee ground emesis [24,25], and, in some cases, stools mixed with life-red blood [26,27]. General symptoms are also noted, i.e., a decrease in blood pressure and an increase in heart rate, which can lead to weakness, anemia, and, in the most serious cases, shock and even death [25].

Indeed, the generally considered boundary between the upper and lower gastrointestinal tract is the ligament of Treitz, also called the duodenal hinge ligament, which is another reason for the presence of upper gastrointestinal bleeding (UGIB) symptoms when the source of bleeding is the damaged liver [28,29,30,31]. 

The cases described of post-traumatic liver parenchymal injury manifested at least partially by UGIB are both from before and after the introduction of advanced imaging methods (scintigraphy, USG, and CT). This prompted the authors to look at the issue in terms of the feasibility of diagnosing blunt liver injury and the impact of imaging studies on subsequent patient outcomes.

## 2. Material and Methods

This research was performed based on described cases of liver injury following blunt trauma manifested dominantly or accompanied by UGIB, where the bleeding originated from the ruptured liver parenchyma rather than other sources, such as the cystic artery bleeding into the biliary tract.

This study was retrospective, monographic in nature and was conducted in tabular descriptive form. The following research methods were used:

- Desk research method;
- Individual case study method;
- Method of analysis and critique of the literature;
- Method of analysis and logical construction;
- Descriptive method;
- Comparative method (according to time criteria);
- Statistical methods.

All cases were found in the following databases: PubMed, Google Scholar, Academic Search Ultimate (EBSCO), Elsevier Journals, BMJ Journals, Springer, Scopus, and Google.

The only selected cases were those in which symptoms of UGIB were present as follows:

- At the time of the patient’s admission to the hospital;
- Before the laparotomy (whether it was performed soon after the patient’s admission to the hospital or at a more distant date, even during subsequent hospitalizations);
- In non-operated patients before their death.

Only cases in which anatomical liver damage was confirmed at either laparotomy or autopsy were retrieved.

We encountered 13 cases from 1848 to 2019. The first group consisted of 7 cases from the era prior to the introduction of scintigraphy (early 1950s), USG (late 1950s), and CT (early 1970s). The period before the introduction of the first of the modern imaging tests (scintigraphy) was taken as the upper time limit for this group (the last case: 1949). The second group consisted of 6 patients related to the cases from the period when all foregoing radiological procedures were already used and widely available. The starting point in this group was after the introduction of the last of the modern imaging tests (CT, starting from 1971).

All cases from both groups are summarized in Table 1 and Table 2 (the first group) and Table 3 and Table 4 (the second group).

These studies’ inclusion criteria, distinguishing cases from two time periods, helped to make the issues of the research problem addressed in the article clear and unambiguous. Group I presented the clinical situation without any interference from modern imaging studies. Group II, on the other hand, presented the impact of these examinations on the fate of patients with as little interference from other factors (e.g., interventional radiology examinations) as possible. Naturally, it was taken into account that general medical advances, including surgical techniques, may have favorably influenced treatment outcomes in the latter group. However, the strict selection of cases made it possible to distinguish two study groups allowing for a meaningful comparative analysis.

The tables include age, gender, circumstances of injury, the presence of UGIB symptoms, the preoperative and intraoperative diagnosis of liver and other organ damage, treatment and clinical outcome, right lower abdominal pain, UGIB, jaundice, complete Quincke’s triad, days from trauma to the onset of UGIB symptoms and indication for the laparotomy. In addition, the authors included the post-mortem diagnosis in Table 1 and radiological diagnosis (by ultrasound, scintigraphy/liver scan, or CT) in Table 3. For the purpose of providing better clarity to the cases analyzed, each group is described in two tables. Cases of patients treated with angioembolization (who, due to the timeframe, would have been included in Table 3 and Table 4) were not taken into the study. The aim was to preserve the comparability of the two groups as much as possible, and the authors intentionally skipped a radiological invasive procedure that may have a significant impact on patient survival. This made it possible to compare the groups treated only surgically, bearing in mind the progress that has been made in surgical techniques over the period under analysis. This allowed us to expose the impact of modern imaging methods on patient’s outcomes, while omitting angioembolization as a factor that can significantly affect patients’ survival. Moreover, angioembolization for obvious reasons was unavailable in the oldest cases (qualified in Table 1 and Table 2).

In addition, only cases in which at least one of the above imaging studies was performed were included. Cases in which these examinations were not available in practice at the hospital in question, although they were already known, were not included. These cases, due to the lack of practical availability of imaging examinations, could have been indeed included in group 1.

## 3. Results

This study included 13 cases from 1848 to 2019, 7 of them are from the period before the introduction of modern imaging methods (early 1950s) and 6 date from when it was already in use (early 1970s) [32,33,34,35].

In the era before scintigraphy, USG, and CT were introduced, there were seven patients, two of them had been surgically treated and five had been treated conservatively. Five patients died, all of them had been managed conservatively. All deceased patients had autopsies that confirmed liver damage as the underlying origin of bleeding. Two patients survived, both of them were treated surgically. In this group, the only surviving patients were those who had undergone surgical treatment. One patient was misdiagnosed with gastric ulcer when the actual source of bleeding was a ruptured liver. This patient was treated by laparotomy and survived. The second surviving patient was operated on due to the presence of symptoms suggestive of intra-abdominal injuries, but without a definite diagnosis of liver rupture. Complete Quincke’s Triad was observed in three of seven patients. The time from trauma to the onset of symptoms with UGIB ranged from 4 to 77 days (11 weeks).

In the second group, all six patients recovered. Three patients were surgically treated, and three were treated conservatively. Complete Quincke’s Triad was observed in two of six patients. The time from trauma to the onset of UGIB symptoms ranged from 6 to 30 days. One patient had only scintigraphy performed, two patients had only CT, and three patients had two examinations (CT and USG).

Due to insufficient data on the age and gender of patients, it was impossible to conduct a precise analysis and calculation of the age structure.

## 4. Discussion

Liver injury following blunt trauma is relatively common, occurring in 1 of 20 patients admitted to the accident and emergency department (A&E) [36]. In contrast, one of four patients with blunt abdominal trauma develops hepatic damage, confirmed by CT scans [37].

Liver injuries following blunt trauma may present as hemobilia with signs of upper gastrointestinal bleeding. The triad of symptoms occurring with hemobilia was first described by Quincke in 1871 and are jaundice, right lower abdominal pain, and upper gastrointestinal bleeding [18]. However, it should be particularly noted that it occurs in only 22–35% of patients with hemobilia [17,21,23]. In the research material, the complete triad of symptoms occurred in 5 of 13 patients. However “Quincke’s incomplete triad” occured in eight cases: right lower abdominal pain and UGIB symptoms were present in five cases and only UGIB signs in three.

Imaging studies other than X-ray play an important role in the diagnosis of traumatic blunt liver injuries and treatment decisions. The first descriptions of the use of scintigraphy were published in the early 1950s (used for kidney diagnostics rather than liver) [32]. Ultrasound (USG) was introduced in the late 1950s [33], and CT in the early 1970s [34,35]. It should also be noted that the timeframe adopted in this study was approximate, since the various new radiological techniques in each country were not introduced simultaneously. No single case described in Table 3 and Table 4 resulted in a patient’s death, and the imaging studies performed (scintigraphy, USG, CT) allowed for the detection of liver injury in each case.

The most used modern radiological procedure was CT (five/six patients, three cases accompanied by USG), and in each case, liver injury was detected. In the era of modern imaging methods, some patients with liver injuries manifesting as UGIB were successfully treated with angioembolization for years [38,39,40]. Angioembolization can replace classical surgical treatment and, in correlation with modern imaging studies, have a positive impact on patient fate. Certainly, there would have been more patients in the second group if angioembolization had not been introduced. Nowadays, the general availability of USG in the emergency department leads to the fact that any patient following a relatively mild abdominal trauma can easily have this test performed (FAST-Focused Abdominal Sonography in Trauma), which can detect liver injury.

It is interesting to point out that the case described by Poulos concerning the incident of 1964 is related to impact on the bicycle handlebars [41]. Symptoms of shock, but no UGIB were present, so they proceeded with a quick exploratory laparotomy with suspected traumatic rupture of the liver or spleen. Symptoms of upper gastrointestinal bleeding did not arise until after a laparotomy, and the patient required a second one. Damage to the parenchyma and serosa of the liver was confirmed during surgery. The patient was operated on immediately and finally recovered. In this case, ultrasound and scintigraphy were not used even though there were first reports of these imaging procedures.

The implementation of imaging studies other than X-ray have been significantly facilitating the diagnosis of patients with UGIB in the course of blunt hepatic injury. This can contribute to quicker and sooner implementation of medical therapy. In the group from the period before the introduction of modern imaging studies, there were only two of seven patients who had been operated on; however, only one had abdominal and general symptoms that could have suggested liver injury (colicky pain and shock) as an indication for surgery [6]. In contrast, in the group from the period after the introduction of modern imaging, the decision to perform laparotomy was determined primarily by radiological imaging (and not according to acute abdominal symptoms), and there is no report that the clinical symptoms noted on physical examination were the key indication for laparotomy [12,13,14]. This is because all patients were operated on at a distant time after trauma [12,13,14]. The shortest time from injury to surgery was 1 month.

It should be pointed out that in the old-period group, only patients who had not been treated surgically and whose liver injury had not been preoperatively diagnosed died [5,7,8,9,11]. On the other hand, those patients who had been operated on survived, although they had not been definitely and accurately diagnosed with liver injury before surgery [6,10]. Failure to undertake surgical treatment in practice ultimately determined the fate of the patients. There is, of course, no certainty that carrying out such treatment would have saved the patients’ lives. However, in knowing the results post mortem, it is possible to conclude that the failure to diagnose the liver injury and the consequent failure to undertake surgical treatment reduced the patients’ already poor chances of survival. However, it should be borne in mind that decisions on surgical treatment were difficult in the face of ambiguous abdominal symptoms or the absence of such symptoms with marked signs of UGIB. It is also important to note that in patients who survived, i.e., underwent surgical treatment, the decision for laparotomy was made without any evident suspicion of liver injury. Therefore, it can be argued that if more advanced imaging studies had been available at the time, they could have helped to establish the true nature of the clinical problem constituting the indication for laparotomy. Indeed, the supravital diagnosis of the liver injury could have influenced the decision on surgical treatment. On the other hand, it should be noted that in the later group, all patients who were not treated with surgery survived. This cannot be explained by the performance of imaging studies only. It is likely that the extent of liver injury was minor. Modern diagnostic techniques have certainly made it easier to monitor and manage patients’ conditions. However, it should be emphasized once again that in the earlier group, the performance of such radiological tests (which were not available in practice) could have made it possible to detect liver injury and thus to decide on surgical treatment. Consequently, this could have increased the patients’ chances of survival. The above observations underscore the beneficial role of modern imaging techniques.

Nowadays, it is possible that liver damage is visualized on imaging studies, but symptoms of UGIB will occur later [13]. This is very important. If symptoms of UGIB occur later, it will not be entirely unexpected, and the most likely reason for the hemorrhage will already be known. In such a context, making further therapeutic choices is considerably easier. It is also impossible to assign a crucial role to the onset of jaundice, which may occur at a long period of time away from the trauma, even a month later [13]. Moreover, it must be remembered that in the event of significant and heavy bleeding, the symptoms of shock may appear quickly and contribute to the decision to proceed with surgery before jaundice is manifested.

Some bleeding localized external to the gastrointestinal tract may have symptoms similar to UGIB and may be a consequence of trauma. As a result of facial trauma with profuse nose bleeding, large amounts of blood may be swallowed, which can be expressed as apparent UGIB, or hematemesis and melena, which occurs in more than half a percent of cases [42].

A mask of upper gastrointestinal hemorrhage can also follow cranial base fracture. Following a temporal bone rupture, blood extravasates into the tympanic cavity and through the auditory (Eustachian) tube enters the lumen of the gastrointestinal tract. This bleeding cause hematemesis that resembles esophageal bleeding [43].

Another example of a rare cause that can mimic UGIB is traumatic rupture of a pharyngeal artery aneurysm due to a car accident. When this happens, blood through the ruptured wall of the branch of the internal maxillary artery escapes into the gastrointestinal tract and manifests as hematemesis and hematochezia [44].

Post-traumatic liver bleeding can also be mimicked by lower gastrointestinal bleeding (LGIB), which accounts for only 20% of all can also be imitated by LGIB, occasionally resulting in black, tarry stools or stools colored with fresh red blood [45]. However, much more often the bleeding is not visible macroscopically and is therefore detected by a fecal occult blood test [46].

Patients with signs of UGIB from whom it is not possible to take a history or determine their fate in the period immediately before being brought to hospital (due to drug or alcohol intoxication or unconsciousness) require special care. It may be advisable to perform FAST even before endoscopy. Such a management would allow a preliminary assessment of the liver for traumatic damage as a cause of UGIB.

It is worth noting that the study showed that endoscopy did not detect all gastrointestinal bleeding and, if it did, it did not necessarily identify the liver as the source of bleeding. In one patient, endoscopy performed at the initial stage proved to be a false negative—it did not show bleeding. Only a repeat endoscopy performed at a later date allowed for the confirmation of bleeding from the papilla of Vater [13]. This case shows that modern diagnostic techniques also do not always guarantee immediate and unambiguous results. In the second patient, endoscopy showed the presence of bulky clots in the stomach and fresh blood in the duodenum, but it was not possible to confirm that the source of the bleeding was the liver [14]. In the third patient, endoscopy did not find any oeso-gastroduodenal lesion in a patient with symptoms of UGIB (hematemesis, hematochezia) [16].

Nevertheless, it needs to be emphasized that the research groups, due to the very restrictive classification criteria for cases (especially the presence of symptoms of UGIB before surgical treatment and the exclusion of cases treated with angioembolization), are small in number, and no categorical conclusions can be drawn on this basis. It is a fact, however, that no fatal cases have been found in the analyzed material since the introduction of modern radiological techniques. This tendency seems to be logical. Obviously, it is not possible to conclude that this is solely a result of the implementation of these examinations into clinical practice, as new treatment techniques, including surgical ones, have a major role to play. Above all, radiological techniques have an impact on rapid and early diagnosis.

The results of this study show the evolution and change that has occurred in the management of patients with blunt liver injuries manifesting as upper gastrointestinal bleeding. In the old days, in practice, only the finding of abdominal symptoms and those associated with blood loss led to a decision for surgical treatment. In contrast, symptoms of upper gastrointestinal bleeding without other clearly marked complaints may have been a confounding factor for doctors and distanced them from the diagnosis of the underlying problem, i.e., liver rupture. Nowadays, on the other hand, imaging studies can detect existing liver injury even before the onset of upper gastrointestinal bleeding symptoms. This significantly speeds up diagnosis and early treatment, which can already be carried out not only surgically, but also by interventional radiology methods.

## 5. Conclusions

1. Liver injury should always be considered as a reason for symptoms of upper gastrointestinal bleeding following abdominal trauma, even if the patient has not suffered a significant abdominal trauma or it cannot be excluded.
2. A liver rupture manifesting as UGIB often presents without the complete Quincke’s triad (less than half of the cases in each group), which may occur following a relatively minor blunt trauma (i.e., impact on bicycle handlebars, fall while running on a curb) and may manifest itself even over 10 weeks after the injury.
3. Before the introduction of USG, scintigraphy, and CT, diagnosing post-traumatic liver injury manifested by UGIB was difficult and carried out with noticeable mortality risk.
4. The introduction of modern imaging methods has made it easier to diagnose liver injuries and decide on surgical or radiological treatment.
5. In patients with symptoms of UGIB, especially those with an unknown or unidentifiable history (e.g., in limited contact, drunk, unconscious), but also with a history of abdominal trauma, it is reasonable to perform FAST already in the hospital emergency department and even before endoscopy to assess the liver for traumatic injury as a potential source of bleeding.

## Figures and Tables

**Table 1:** Patients from the period before the introduction of modern imaging tests—basic overview.

No	Case	Age	Sex	Trauma	PD+	C/L*	Intraoperative Diagnosis	D/R#	Post-Mortem Diagnosis
1	Owen (1848) [5]	22	M	Falling out of the carriage	-	C	No laparotomy	D	Extensive rupture on the surface of the right hepatic lobe
2	Siegel (1909) [6]	32	M	Impact on bicycle handlebars	-	L	A few liver ruptures, blood clots, gallbladder gangrene, and mild hemoperitoneum	R	-
3	Strauss (1929) [7]	19	M	Hitting a tree with sled	-	C	No laparotomy	D	Liver rupture with blood reservoirs hepatic and subdiaphragmatic reservoirs communicating with bile ducts, hemoperitoneum.
4	Thorlakson Hay (1929) [8]	45	M	Fall on a woodpile	-	C	No laparotomy	D	Numerous partially healed ruptures of the right hepatic lobe, with necrosis inside, haemobilia
5	Wulsten (1931) [9]	11	F	Hitting the fence with a sled	-	C	No laparotomy	D	Cavity the size of a “child’s head” in the right liver lobe filled with blood clots and reddish-brown fluid, liver sac intact
6	Hawthorne (1941) [10]	?	M	Being hit by a car rim during an accident	Pyloric defect interpreted as gastric ulcer on X-ray	L	Enlarged gallbladder, with an oval mass consisting of bile and clots	R	-
7	Drescher (1949) [11]	2	?	Being kicked by horse	-	C	No laparotomy	D	Adherent clot-like masses 2.5 × 1.5 cm on the diaphragmatic surface of the liver closely. Two parallel lacerations 2 cm long on the visceral surface of the right hepatic lobe. Two capsular tears penetrating the liver parenchyma to a depth of 0.5 cm and a large irregular laceration on the border of both liver lobes, creating a cavity filled with clots. Several small cavities filled with blood near the above-described cavities.

*C/L—conservative management (C) or laparotomy treatment (L). #D/R—death or recovery. PD+—preoperative diagnosis of liver or other organ injury.

**Table 2:** Patients from the period before the introduction of modern imaging tests—detailed characteristics of symptoms.

No	Case	Right Lower Abdominal Pain	UGIB	Jaundice	Complete Quincke’s Triad	Days from Trauma to the Onset of UGIB Symtomps	Indications for the Laparotomy
1	Owen (1848) [5]	+	Melena Hematochezia	+	+	5	No laparotomy
2	Siegel (1909) [6]	+	A large coagulum of blood by rectum (deemed to be of hepatic origin)	+	+	10	Colicky pain, shock 14 days after trauma, exploratory laparotomy
3	Strauss (1929) [7]	-	Melena	-	-	12	No laparotomy
4	Thorlakson Hay (1929) [8]	+	Melena	+	+	8	No laparotomy
5	Wulsten (1931) [9]	+	Melena Hematochezia Hematemesis	-	-	21	No laparotomy
6	Hawthorne (1941) [10]	+	Melena Hematochezia Hematemesis	-	-	77	Suspected gastric ulcer
7	Drescher (1949) [11]	+	Hematochezia Hematemesis	-	-	4	No laparotomy

**Table 3:** Patients from the period after the introduction of modern imaging tests—basic overview.

No	Case	Age	Sex	Trauma	Preoperative Diagnosis of the Liver or Other Organs Injuries	Use CT/USG/SC	C/L/E*	Radiological Diagnosis (Confirm on USG/CT/SC)	Intraoperative Diagnosis	R/D#
1	McGehee (1974/1972) [12]	28	M	Car accindent	+	+ SC	L	Hepatic arteriogram- an avascular mass in the center of right hepatic lobe with a pseudoaneurysm 2.5 × 3.5 cm in diameter. Liver scan: lesion between right and left hepatic lobes.	Hepatomegaly, firm mass bulging from the superior portion of the anterior surface of the right hepatic lobe.	R
2	Bajpai (1989/1976) [13]	11	M	Blunt abdominal trauma	+	+ CT	L	Liver scan showed a space-occupying lesion in the anterosuperior part of the right liver lobe. Endoscopy showed fresh blood flow from the ampulla of Vater. Angiography showed extravasation of dye in the area corresponding to the space-occupying lesion.	A large hematoma was evacuated from the right liver lobe, and a bleeding vessel was transfixed and ligated with silk sutures. An area of bile leakage was also transfixed and ligated with chromic catgut sutures. The liver cavity was drained with a Malecot catheter.	R
3	Wani (2011) [14]	12	M	Car accident	+	CT (2) and USG	L	Defect in the right hepatic lobe of the liver with adjacent perihepatic fluid collection, enhancing lesion anterior to the right branch of the portal vein. USG: fluid collection around the right lobe of the liver; hepatic bile ducts were slightly Dilated.	Extensive damage to the right hepatic lobe.	R
4	Bardes (2011) [15]	13	M	Impact on bicycle handlebars	+	CT (3)	C	Liver damage with intrahepatic hematoma, gallbladder filled with clots. Angiogram: pseudoaneurysm of right hepatic artery without extravasation contrast, slight dilatation of intrahepatic bile ducts.	-	R
5	Jalal (2019) [16]	36	?	Abdominal trauma	+	CT (2) and USG	C	Ultrasound and initial CT showed poorly limited hyperechoic lesions of segments IV, V, VII, and VIII related to foci of bruising. The 2nd CT showed, in addition to hepatic contusion, a dilatation with hemorrhage of the intrahepatic bile ducts and gall bladder.	-	R
6	Jalal (2019) [16]	53	?	Polytrauma with abdominal impact	+	CT (3) And USG	C	Ultrasound and 1st CT showed fracture of the IV segment of the liver with contusions of segments VI and VII. The 2nd CT-peri-hepatic peritoneal effusion on segment IV of the liver. The gallbladder had an intra-mural hematoma. The 3rd CT scan showed a large biliary collection of the liver extending from the para-colic gutter to the right iliac fossa.	-	R

*C/L/E means conservative treatment or laparotomy or embolization. #R/D means recovery or death.

**Table 4:** Patients from the period after the introduction of modern imaging tests—detailed characteristics of symptoms.

No	Case	Right Lower Abdominal Pain	UGIB	Jaundice	Complete Quincke’s Triad	Days from Trauma to the Onset of UGIB Symtomps	Indications for the Laparotomy
1	McGehee (1974/1972) [12]	+	Melena Hematemesis	-	-	14	Following the radiological diagnosis suported by angiography (liver lesion)
2	Bajpai (1989/1976) [13]	+	Melena Hematemesis	+	+	30	Following radiological diagnosis suported by angiography (liver lesion)
3	Wani (2011) [14]	-	Melena Hematemesis	-	-	30	Following the radiological diagnosis (hepatic laceration)
4	Bardes (2011) [15]	+	Hematemesis	-	-	6	No laparotomy
5	Jalal (2019) [16]	+	Hematemesis Hematochezia	-	-	8	No laparotomy
6	Jalal (2019) [16]	+	Hematemesis	+	+	6	No laparotomy

## Data Availability

Not applicable. The study was a pooled one, and the clinical cases used for the study were taken from those previously described in the professional literature. Each of the scientific articles used was cited and described in detail.
